# Carboplatin Is Associated with Changes in Components of the m^6^A Machinery in Triple-Negative Breast Cancer Cells

**DOI:** 10.1021/acsomega.6c01546

**Published:** 2026-07-07

**Authors:** Ricardo Villalobos-Valencia, Carlos R. Alvizo-Rodríguez, Alan Carrasco-Carballo, José A. Sierra-Ramírez, Uriel López-Vázquez, Emmanuel Seseña-Méndez, Marta E. Hernández-Caballero

**Affiliations:** † Sección de Estudios de Posgrado e Investigación, Escuela Superior de Medicina, 27740 Instituto Politécnico Nacional, Ciudad de México 11340, México; ‡ Laboratorio de Biología del Cáncer, Facultad de Medicina, Biomedicina, 103612 Benemérita Universidad Autónoma de Puebla, Puebla 72410, México; § Secretaría de Ciencia, Humanidades, Tecnología e Innovación, Laboratorio de Elucidación y Síntesis en Química Orgánica, Herbario y Jardín botánico, Vicerrectoría de Investigación y Estudios de Posgrado, BUAP, Puebla 72410, México; ∥ Laboratorio de Neuroinmunologia, Facultad de Medicina, Biomedicina, 103612 Benemérita Universidad Autónoma de Puebla, Puebla 72410, México

## Abstract

Triple-negative breast cancer (TNBC), a breast cancer subtype characterized by aggressiveness and high recurrence, is usually treated with chemotherapy agents, such as platinum-based drugs. Chemotherapy modulates gene and protein expression, including epitranscriptomic regulators such as components of the m^6^A methylosome. This study examined the effects of carboplatin (CP) on the expression of m^6^A methylosome machinery (METTL3, METTL14, WTAP, FTO, YTHDF2) and the m^6^A methylation mark in two cell lines (HCC1937 and MDA-MB-231). Using KM-plotter, a survival analysis was performed, while molecular docking and molecular dynamics (MD) simulations were conducted to identify critical structural interactions between CP and m^6^A methylosome components. Gene and protein expression analyses revealed that FTO was consistently downregulated after CP treatment in both cell lines. Bioinformatic survival analysis linked low FTO expression to improved patient survival, and in silico docking and MD simulations confirmed that FTO established the most stable interaction with CP. Conversely, METTL3 expression was decisive for the global m^6^A methylation mark, being upregulated in HCC1937 cells (increasing m^6^A marks) but downregulated in MDA-MB-231 cells (decreasing m^6^A marks), while high METTL3 transcripts correlated with better prognosis. Docking and MD insights suggested strong interactions between CP and FTO/METTL3 proteins. These findings indicate that CP influences m^6^A machinery in TNBC cells, potentially affecting epitranscriptomic regulation and chemotherapy response.

## Graphical abstract

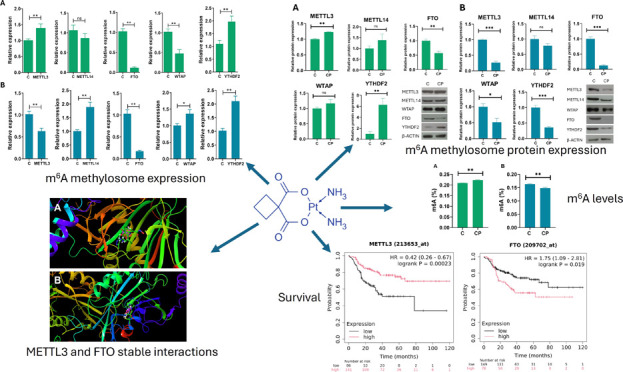

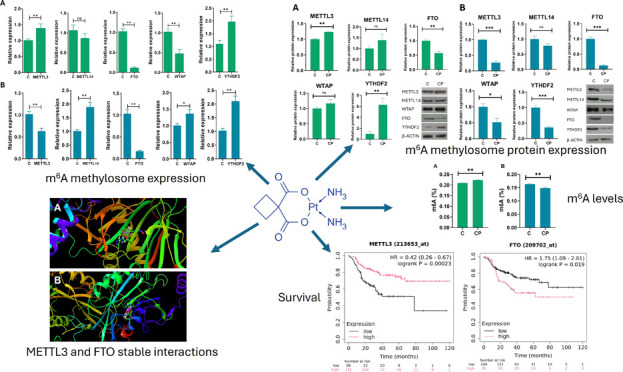

## Introduction

Triple-negative breast cancer (TNBC) accounts for about 15–25% of all breast cancer cases.^1^ TNBC lacks estrogen and progesterone hormone receptor (ER and PGR, respectively) expression in addition to human epidermal growth factor receptor (HER2) expression and is more often found in young women. This cancer has a higher rate of metastasis and mortality, which are linked to its propensity for relapses. The absence of these receptors leads to insensitivity to hormone and HER2 therapies, thus dictating that chemotherapy is the main treatment option. Patients’ median overall survival is 10.2 months.^2^

Conventional treatments for TNBC include surgery followed by adjuvant chemotherapy, but this regimen depends on the state of the tumor in each patient. Carboplatin (CP), pembrolizumab, and paclitaxel regimen is the current standard in preoperative therapy.^3^ CP is a second-generation analog of cisplatin and possesses reduced systemic toxicity (but induces myelosuppression), which allows its use as high-dose chemotherapy for aggressive tumors, such as ovarian, lung, head, and neck cancers. CP forms adduct CP–DNA by covalently binding to the N7 of guanine to yield inter- and intrastrand cross-links. These structures affect both the double helix and nucleosome conformation and cause an increase in DNA synthesis, transcription, and cell cycle blocks in addition to facilitating apoptosis due to extensive DNA damage.^4,5^ However, CP eventually produces drug resistance that is caused by an increase in the DNA mismatch repair process.^5^ BRCA1 is a tumor suppressor, which, together with BRCA2, is involved in DNA repair. Mutations in the BRCA1 gene predict susceptibility to high-risk breast, ovarian, and other cancer types.^6^ Breast cancer with BRCA1 mutations has a more aggressive phenotype, is found in 10–20% of TNBC cases and is usually associated with shorter overall patient survival. BRCA1/2-deficient cells are more sensitive to platinum chemotherapy due to a defect in homologous recombination DNA repair.^7^

Epigenetic modifications are alterations that are considered one of the hallmarks of cancer. *N* ^6^-methyladenosine (m^6^A) is the most studied epitranscriptomic RNA modification and is involved in many biological processes. Its functions are mediated by writer, eraser, and reader proteins.^8^ Methyltransferase-like-3 (METTL3) is an RNA methyltransferase that forms a heterodimer with METTL14 and a complex with Wilms tumor 1-associating tumor (WTAP), RNA binding motif protein 15 (RBM15), Hakai protein (CBLL1), zinc finger CCCH-type containing 13 (ZC3H13), and vir-like m^6^A methyltransferase-associated (VIRMA). This writer complex is responsible for adding the m^6^A modification. As with other epitranscriptomic modifications, m^6^A is dynamically added and removed and is eliminated by the eraser enzymes, fat mass and obesity-associated protein (FTO), and demethylases alkB homologue 5 (ALKBH5). Furthermore, m^6^A can be bound by nuclear YT521-B homology domain-containing proteins (YTHDC1/2) and cytoplasmic YTH m^6^A RNA-binding proteins (YTHDF1–3) acting as reader proteins.^9^ Based on the results of various studies, it has been found that expression dysregulation in subunits of the methylosome complex alters the sensitivity of cancer cells to chemotherapy. Platinum-based chemotherapy treatments have been reported to modulate methylation at the DNA level, which has been associated with tumoral resistance.^10^

Given that epitranscriptomic RNA modifications have recently emerged as critical drivers of treatment resistance in cancer cells, this study investigated two TNBC cell lines, MDA-MB-231 and HCC1937. We elucidated how CP modulates the expression of core methylosome components—specifically METTL3, METTL14, FTO, WTAP, YTHDF2, and m^6^A levels. By correlating these expression profiles with clinical databases, we analyzed gene expression to understand the broader impact of chemotherapy on epitranscriptomic regulators in breast cancer patients. Finally, the interactions between CP and these methylosome proteins were explored using molecular docking simulations to identify their high -affinity sites.

## Materials and Methods

### Cell Culture

The human breast cancer lines, MDA-MB-231 (adenocarcinoma, normal BRCA1) and HCC1937 (TNM stage IIB, grade 3, primary ductal carcinoma, BRCA1 mutation 5382insC), were cultured in Dulbecco’s Modified Eagle’s Medium (DMEM, Gibco, Thermo Fisher, USA) and RPMI 1640 (Gibco, 31800-022, Thermo Fisher, USA), respectively. The medium was supplemented with 100 U/mL penicillin, 100 μg/mL streptomycin, 0.25 μg/mL (Gibco, Thermo Fisher, USA), and 10% (v/v) fetal bovine serum (FBS, Biowest, S1400-500, Fr). The cells were incubated at 37 °C in a 5% CO_2_ atmosphere.

### MTT Assay

To estimate the cytotoxic effect of the carboplatin (CP; injectable solution 450 mg, Nuvaplast, Accord), the MTT (3-[4,5-dimethylthiazol-2-yl]-2,5-diphenyltetrazolium bromide; M6494, Thermofisher Sci, USA) test was performed on the cancer cell lines. MDA-MB-231 and HCC1937 cell lines were seeded in 96-well plates (1 × 10^4^), cells were left 24 h for attachment. The MDA-MB-231 cells were exposed to CP at a concentration range of 0–200 μg/mL, and HCC1937 cells were exposed to CP at a concentration range of 0–70 μg/mL. After exposure to CP for 72 h, MTT (0.5 mg/mL) was added to the cells for 4 h. The absorbance of the formazan solution (in isopropanol and HCl 0.01N) was measured on a microtiter plate reader at 570 nm (Accuris, BioTek Benchmark Scientific, USA). Data were analyzed by logistic dose response, and with this the IC_50_ was obtained. Each dot represents mean values (±SEM). Data represent the mean of three independent experiments; each performed in duplicate.

### Wound Healing Assay

HCC1937 and MDA-MB-231 cells were seeded in 6-well plates and incubated until they reached 70% confluence under the initially indicated culture conditions. After confluence was reached, the breast cancer cell monolayers were scratched by a straight line using a 200 μL sterile pipet tip and a grid design to obtain the same field for each image acquisition. After that step, phosphate-buffered saline (PBS) 1X was used to wash cells and remove cell debris. A group treated with CP and an untreated group (control) were included. The photographs of the scratch wound were recorded every 24 h (0–72 h) to score cell migration. Digital photographs were obtained using an inverted microscope on a 4X objective (Motic AE2000, Ca). The scratch area was determined using the Image-J software as the percentage of area reduction (wound closure).^11^ All experiments were performed in triplicate, and results were expressed as mean ± SEM.

### RT-qPCR

Total RNA was extracted from breast cancer cell lines with TRIzol reagent (Invitrogen, 15596026, USA) according to the manufacturer’s instructions. After extraction, the quality and quantity of the total RNA were measured using the Nanodrop system (Thermo Fisher Scientific, USA). cDNA was synthesized using the Accuris qMax First Strand cDNA Synthesis Flex Kit (Accuris, USA) with 1 μg of total RNA. The cDNA was used as a template for the Kapa SYBR Fast qPCR Master Mix (2X) kit (SF1UKB, MilliporeSigma, USA) for gene expression analysis. The transcript expression levels were measured using the Step One real-time PCR system (Applied Biosystems USA). The relative quantity or fold change in the target genes was then normalized to the level of hypoxanthine phosphoribosyltransferase 1 (HPRT1) and to a control group (untreated cultured cells). Each reaction was performed in triplicate, and data were analyzed with delta delta Ct method. All real-time polymerase chain reaction (qPCR) primers were designed using Primer3 and BLAST from the NCBI and were synthesized by Integrated DNA Technologies (IDT, USA). The primer sequences are shown in Table 1.

**Table 1:** PCR Primer Sequences

**primers**	**sequences (5′-3′)**	**PCR product sizes**
**FTO**		
F	CAGTGAGCGAGGCAAGGATG	132
R	TGGCTCAACTGGAAGCACTG	
**METTL3**		
F	TGGAGACAATGCTGCCTCTG	116
R	GCTCTATCCAGGCCCACAAG	
**METTL14**		
F	TTAGGAGCACTGAAATAGGATGC	120
R	ACGCCTTCATCTATTTGGAAGAG	
**WTAP**		
F	AGTTGTGCAATACGTCCCTGG	122
R	AGTGCCTGGAAGTTTACGCC	
**YTHDF2**		
F	CGTTGCTGCAGTCTGTGTAG	133
R	GCTACAAGCACACCACTTCC	
**HPRT1**		
F	GTAATCCAGCAGGTCAGCAA	160
R	TGCTGAGGATTTGGAAAGGG	

### Western Blot

Total protein was extracted from controls and treatment breast cancer cell lines MDA-MB-231 and HCC1937 using TRIzol reagent (Invitrogen, 15596026, USA). The protein levels were quantified using a Pierce Bradford Protein Assay Kit (Thermo Scientific, 23200, USA) in a 96-well plate. Absorbance was measured on a microtiter plate reader at 590 nm (Accuris, BioTek Benchmark Scientific, USA). Proteins were separated via sodium dodecyl sulfate polyacrylamide gel electrophoresis (SDS-PAGE) and transferred onto polyvinylidene fluoride (PVDF) membranes (Merck Millipore, IPVH00010, USA) at 100 V for 1.5 h. PVDF membranes were blocked using 5% nonfat dry milk in Tris-buffered saline (TBS) 1X for 1 h at room temperature followed by incubation with the primary antibodies METTL3 (1:1000; Cell signaling, 96391), METTL14 (1:1000; ABclonal, A8530), WTAP (1:2000; Cell signaling, 56501), FTO (1:500; Cell signaling, 14386), YTHDF2 (1:1000; Cell signaling, 80014), and B-actin (1:2000; Cell signaling, 4967) at 4 °C overnight with gentle shaking. The membranes were washed three times after which the PVDF membranes were incubated with horseradish peroxide (HRP)-conjugated antirabbit secondary antibody (Cell signaling, 7074) for 1 h at 4 °C with gentle shaking. The membranes were incubated with SuperSignal West Pico PLUS Chemiluminescent Substrate (Thermo Fisher, 34577, USA). Hyperfilm ECL (Amersham, 28-9068-35) was then exposed to chemiluminescence for 5–20 min. Finally, the films were exposed to developer and fixer solutions (Carestream, 860, 6899) after which photos were taken and analyzed with ImageJ software.^11^

### Global m^6^A Methylation

Total RNA was isolated from cells as previously described, and the m^6^A RNA Methylation assay kit colorimetric (Abcam, 185912, UK) was used to examine the absolute N6-methyladenosine modification level according to the manufacturer′s protocol. Total RNA (200 ng) from both cell lines in the control and treated groups was added to the strip wells for binding. They were then washed and incubated with the capture antibody, after which m^6^A was detected, and absorbance was read at 450 nm. Each experiment was performed in three independent replicates.

### Survival Assay

Kaplan–Meier Plotter, an online survival analysis tool, was used to correlate methylosome gene expression using mRNA information obtained by gene chip (Kaplan–Meier plotter), with autoselect best cutoff based on expression values. This online tool performs all real-time calculations from a manually curated database. The selected parameters were restricted to several subtypes: (1) ER status negative, (2) PGR status negative, (3) HER2 negative, (4) cohort with systemic treatment by chemotherapy without endocrine therapy, (5) recurrence-free survival (RFS), and (6) follow-up threshold for 120 months. Protein expression analysis was not performed due to a lack of sufficient patients after filtering the data with the required parameters in the database.^12,13^

### Molecular Docking

CP was prepared and optimized at physiological conditions of pH = 7.4 using the OPLS4 force field in the LigPrep module (Schrödinger Release Notes - Release 2025-3), and the Metal was stabilized and simulated using the Jaguar module, both from the Schrodinger-Maestro platform, for molecular docking studies.^14^ The inhibitors of each of the methylosome proteins analyzed were prepared using the LigPrep module according to the previously reported methodology,^15^ (Supporting Information Table S1). The FTO (9KNI), METTL3/METTL14­(6TTP), WTAP (7VF2), and YTHDF2 (7R5L) proteins were obtained from the Protein Data Bank (PDB; RCSB PDB: Homepage) and prepared using water as a solvent and with a hydrogen optimization process and minimized to 0.3 A of RMSD by protein preparation wizard module (Schrödinger Release Notes - Release 2025-3). The couplings were validated by redocking in all cases, obtaining RMSD values less than 2.0 in all cases. Docking was performed in the Glide module (Schrödinger Release Notes - Release 2025-3) with extra precision (XP) according to the previously reported protocol, and the docking studies were presented using the docking score as a comparative parameter.^16,17^

### Molecular Dynamics

#### Geometry Optimization and Partial Charges Calculations

Starting from the docked structures, the ligand-protein complexes from molecular were constructed. Due to the presence of the platinum­(II) metal Center, QM-based parametrization was performed using the Metal Center Parameter Builder MCPB.py.^17^ QM calculations were carried out using ORCA 6.1.0 ^18^ at the B3LYP/def2-TZVP level of theory, employing the def2/J auxiliary basis set with the RIJCOSX approximation.^19^ Three successive calculations were performed: (i) geometry optimization of the full carboplatin complex (using TightOpt convergence criteria) to obtain the reference equilibrium geometry; (ii) a numerical frequency calculation (NumFreq) on the optimized geometry to derive harmonic force constants for Pt–N and Pt–O stretching and bending modes; and (iii) a single-point calculation to derive electrostatic potential (ESP)-based charges over the full complex. The total charge of carboplatin was set to 0 (Pt^2+^ + CBDCA^2–^ + 2NH_3_) with a singlet spin state (multiplicity = 1), consistent with the diamagnetic character of square-planar Pt­(II) d^8^ complexes. Finally, the protein–ligand complexes were assembled using the tleap module of AmberTools.^20^ The protein was described with the ff14SB force field while the carboplatin parameters derived from MCPB.py (bonded model, RESP charges, and GAFF2 parameters for the organic fragment) were loaded.^21^

#### MD Simulation

The complexes were solvated in an orthorhombic TIP3P water box with a minimum buffer distance of 12 Å.^22^ The concentrations of Na+ and Cl- were adjusted to 0.15 M to replicate physiological ionic strength. All molecular dynamics simulations were performed using AMBER26 ^23^ on an Nvidia L4 GPU, the system was energy-minimized in four sequential stages: (i) minimization of solvent and ions with positional restraints on the protein and ligand (10 kcal mol^–1^ Å^–2^); (ii) minimization of the ligand with restraints on the protein only (10 kcal mol^–1^ Å^–2^); (iii) minimization with weak restraints on the ligand only (1 kcal mol^–1^ Å^–2^); and (iv) unrestrained minimization of the full system. Each minimization stage consisted of 2500 cycles of steepest descent followed by 2500 cycles of conjugate gradient. Following minimization, the system was gradually heated from 0 to 300 K over 500 ps under NVT conditions using the Langevin thermostat (collision frequency γ = 1 ps^–1^), maintaining positional restraints on the protein and ligand heavy atoms (10 kcal mol^–1^ Å^–2^). Pressure equilibration was subsequently performed for 1 ns under NPT conditions at 300 K and 1 atm using the Monte Carlo barostat, with gradually reduced restraints. Production MD simulations were carried out under NPT conditions for 120 ns without restraints. Electrostatic interactions were handled using the Particle Mesh Ewald (PME) method with a real-space cutoff of 10 Å. Bonds involving hydrogen atoms were constrained using the SHAKE algorithm,^24^ enabling a 2 fs integration time step. Trajectory analyses were performed using the cpptraj module of AmberTools26.^25^ The stability and structural dynamics of the system were assessed through the following metrics: backbone RMSD, ligand RMSD, RMSF, and radius of gyration.

### Statistical Analysis

Data are presented as mean ± SEM. All statistical analyses were performed using GraphPad Prism (GraphPad Software, Inc., San Diego, CA, USA) and OriginPro Student 2025 software. *p* < 0.05 was considered significant.

## Results

### Effect of Carboplatin on Cellular Migration

After HCC1937 and MDA-MB-231 cells were treated with CP, which induces cell death, the MTT assay was used to obtain the CP IC_50_. Cells were exposed to a concentration range of 0–200 μg/mL for 72 h. IC_50_ values were 59 and 125 μg/mL for HCC1937 and MDA-MB-231 cells, respectively (Figure 1).

**Figure 1:**
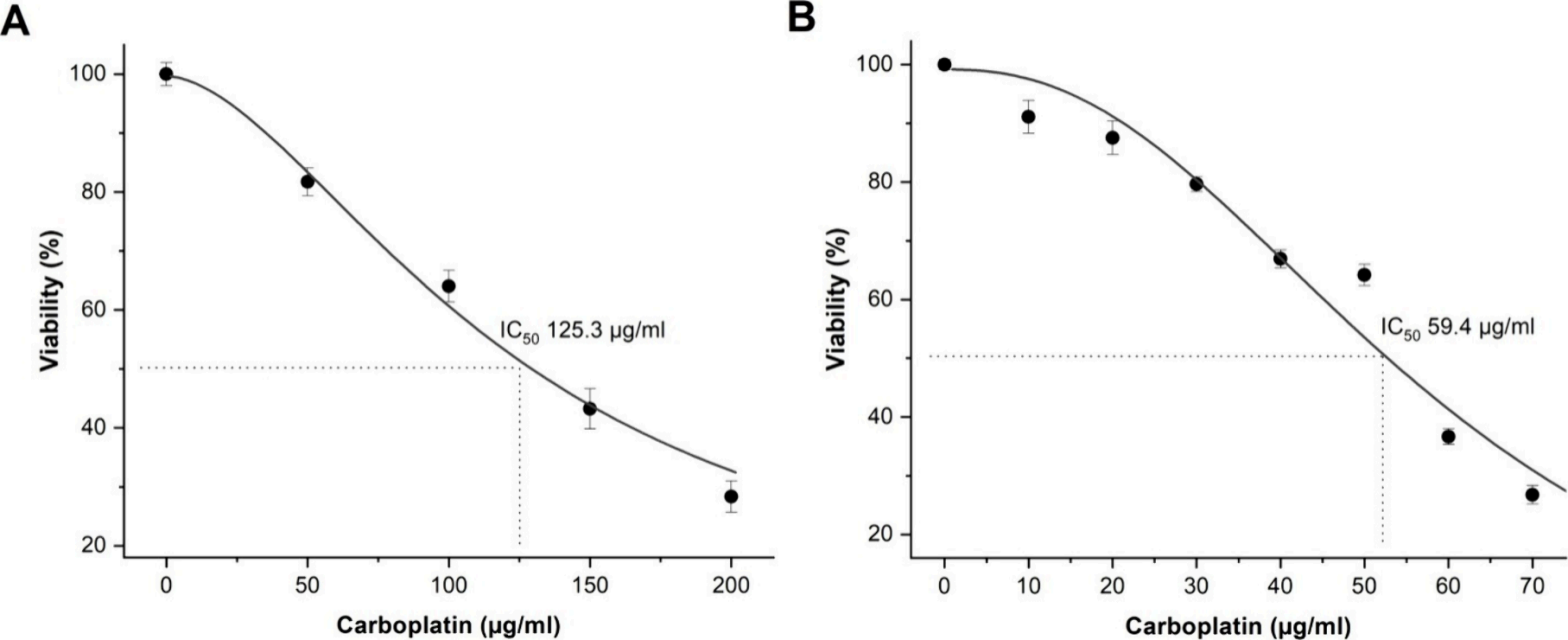
Effect of exposure to CP in MDA-MB-231 and HCC1937 cell lines. Viability of CP-treated and untreated cells after 72 h. Data were plotted for CP (μg/mL) vs viability (%), and the result was obtained with logistic dose response. For (A) MDA-MB-231 IC_50_ was 125 μg/mL, and in (B) HCC1937, it was 59 μg/mL.

Once the appropriate concentration for subsequent experiments was identified, we confirmed the differential inhibitory effect of CP on cell migration in cell lines. The migration capacity was evaluated based on the wound healing assay in both cell lines using the previously determined IC_50_ concentrations (59 and 125 μg/mL, respectively). As expected, CP inhibited movement, and the effect of CP was statistically significant after 48 h in both cell lines. The most noticeable effect was observed in MDA-MB-231 cells. Data were further analyzed using a two-way analysis of variance (ANOVA) with a post hoc Holm–Sidak test, which indicated that the factors of time and CP are significant (Figure 2). Although we do not have evidence of direct binding of CP to cytoskeletal proteins, it has an indirect effect, particularly as an increase in ROS, which, among its consequences, is damage to structural proteins such as actin.

**Figure 2:**
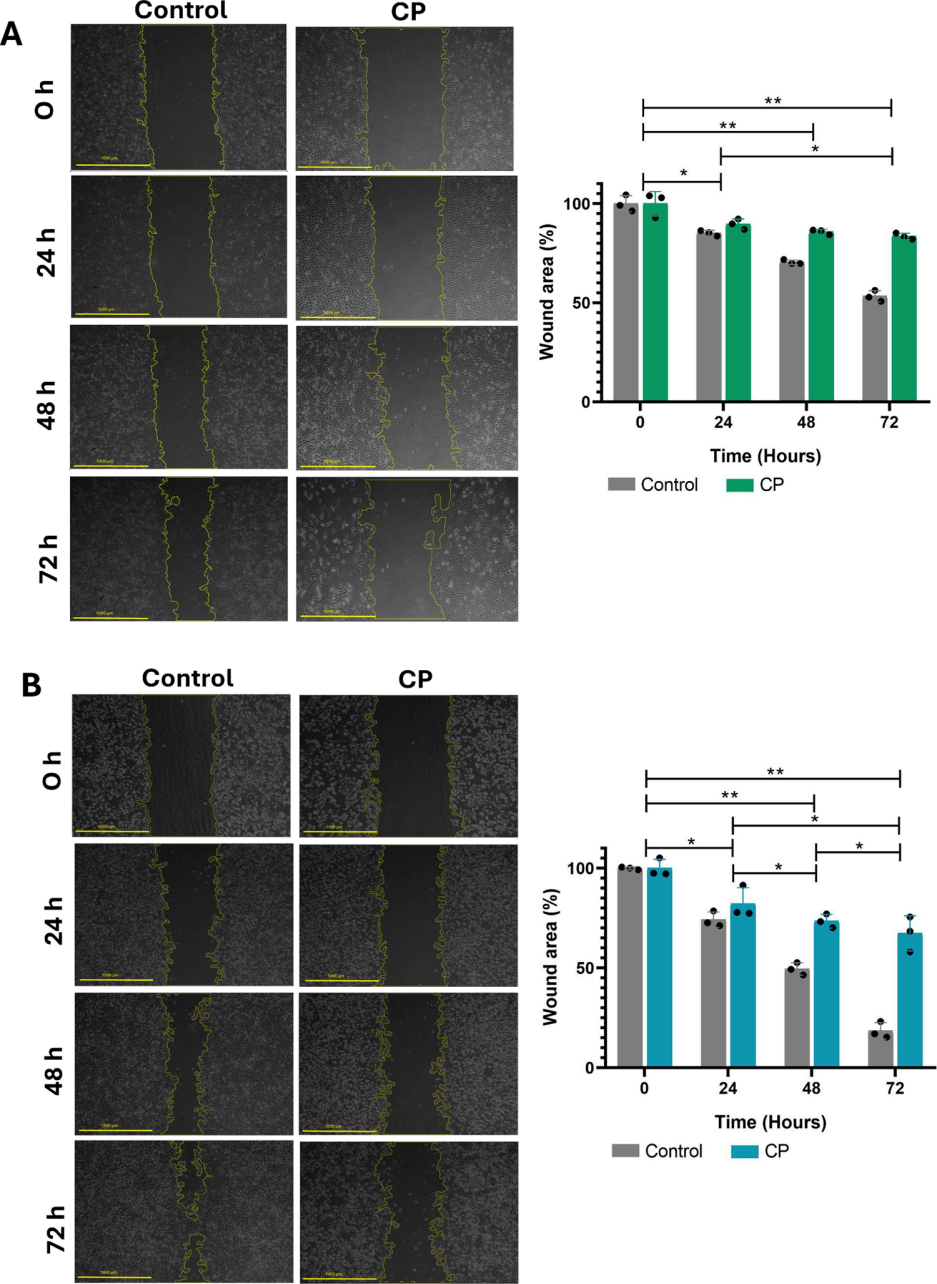
Effect of CP on cell migration. (A) HCC1937 and (B) MDA-MB-231 cells were exposed to CP treatment for 72 h. The effect of CP was evaluated with the wound healing assay using 59 μg/mL to HCC1937 and 125 μg/mL to MDA-MB-231. The statistical significance was determined by calculating the p-value, using two-way ANOVA, the mean ± SEM of percentage cell viability of the human breast cancer cells was compared; *n*: 3. The statistical significance is represented by asterisks (* *p* < 0.05; ** *p* < 0.01), α= 0.05. Bar scale: 1000 μm.

### Methylosome Gene Expression after Carboplatin Treatment

Reverse transcriptase polymerase chain reaction (RT-qPCR) was performed to analyze the effect of 72 h of CP treatment on gene expression in the two cancer cell lines. The results showed a variable impact on cell lines in addition to gene-dependence, and the differences were statistically significant (* *p* < 0.05 and *** *p* < 0.001, respectively). The mRNA expression in HCC1937 cells indicated that FTO and WTAP were down-regulated, while METTL3 and YTHDF2 were up-regulated, but METTL14 showed no differences (Figure 3A). MDA-MB-231 cells showed overexpression of METTL14, WTAP, and YTHDF2, while METTL3 and FTO were down-regulated (Figure 3B). FTO and YTHDF2 exhibited similar responses to CP treatment in both TNBC cell lines.

**Figure 3:**
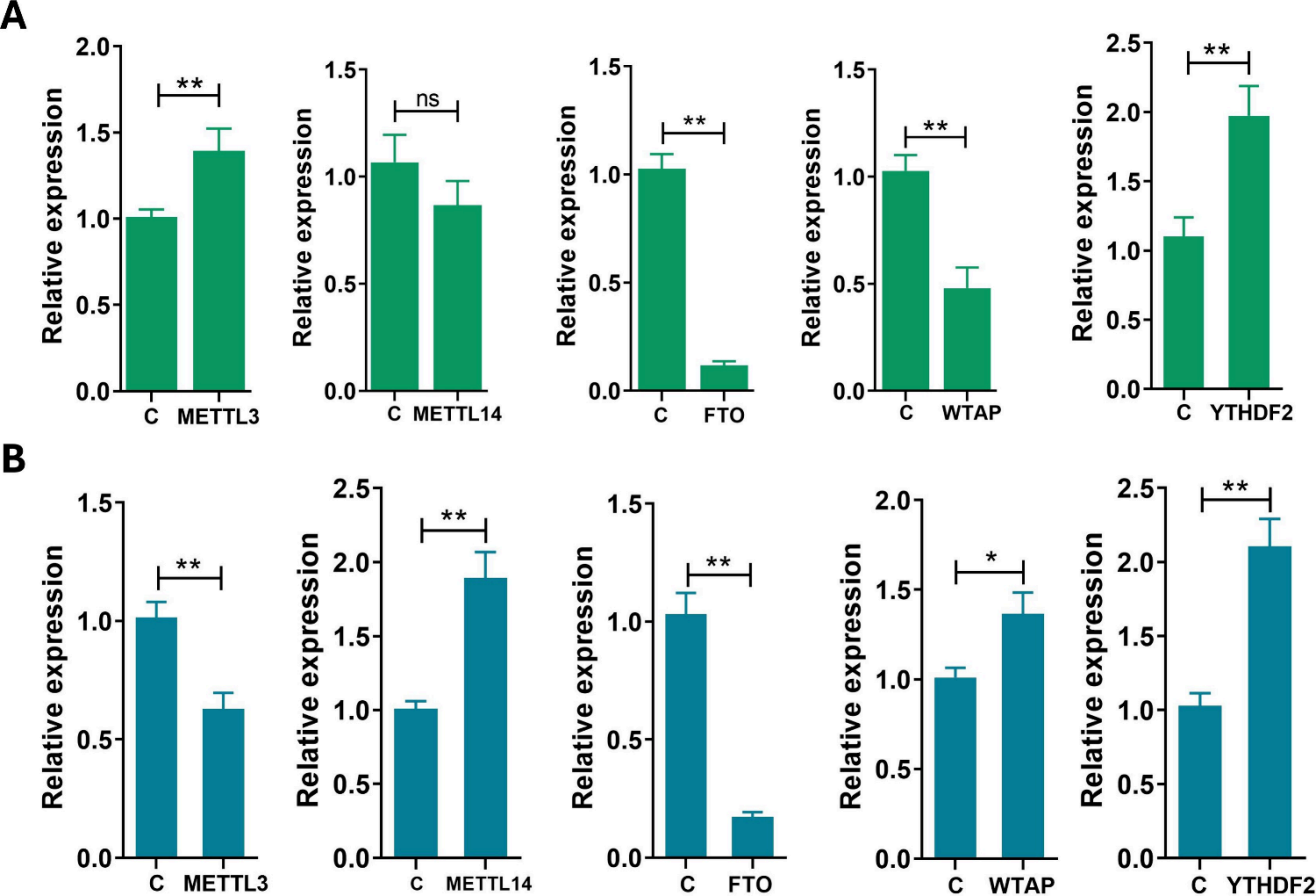
Expression of methylosome genes in cells exposed to CP. RT-qPCR detection of METTL3, METTL14, WTAP, FTO and YTHDF2. (A) HCC1937 cells, (B) MDA-MB-231 cells. The statistical significance is represented by asterisks (* *p* < 0.05; ** *p* < 0.01). ns: no significant (*p* > 0.05); CP: carboplatin.

### Effect of CP on Methylosome Protein Expression

In HCC1937 cells, METTL3 was slightly upregulated, YTHDF2 was upregulated, and FTO was downregulated, but METTL14 and WTAP showed no significant differences (Figure 4A). MDA-MB-231 cells showed subexpression of all proteins; only METTL14 showed no significant differences (Figure 4B). FTO showed the same behavior with CP treatment in both TNBC cell lines.

**Figure 4:**
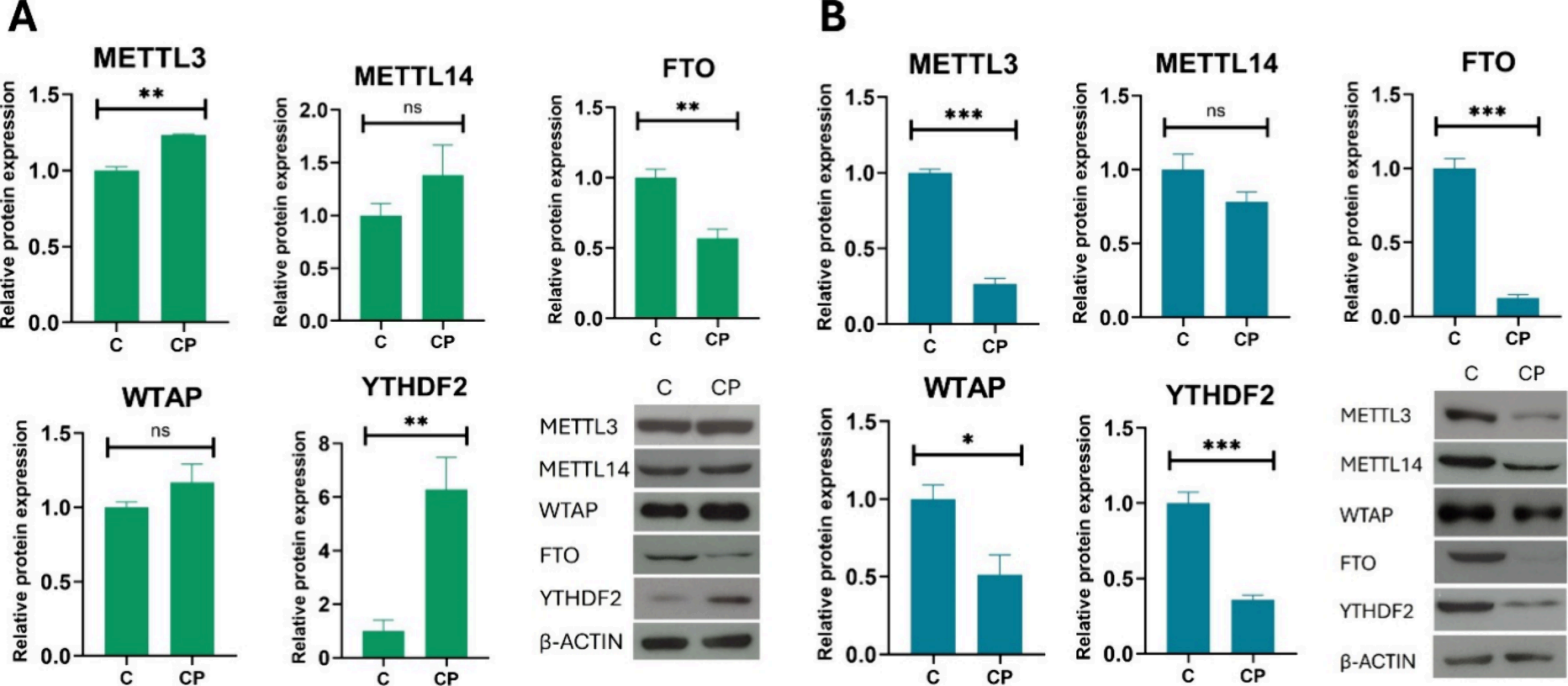
Relative protein expression in cells exposed to CP. Western blot detection of METTL3, METTL14, WTAP, FTO, and YTHDF2. (A) HCC1937 cells, (B) MDA-MB-231 cells. The statistical significance is represented by asterisks (* *p* < 0.05; ** *p* < 0.01; *** *p* < 0.001). ns: no significant; C: control; CP: carboplatin. Three biological replicates were included in the experiment. Data are expressed as the mean ± standard error (SEM). A *t* test was applied for group comparisons.

### Effect of CP on Global m^6^A Methylation

Treatment with CP in TNBC showed opposite results. Increased m^6^A methylation marks were detected in HCC1937, while in MDA-MB-231, methylation marks were reduced compared to their respective controls, thus corroborating the important role of METTL3 as a methyltransferase whose RNA and protein expression were increased in HCC1937 cells and reduced in MDA-MB-231 cells. The increase in HCC1937 and decrease in MDA-MB-231 cells resulted in an increase and decrease in the methylation marks in HCC1937 and MDA-MB-231, respectively (Figure 5).

**Figure 5:**
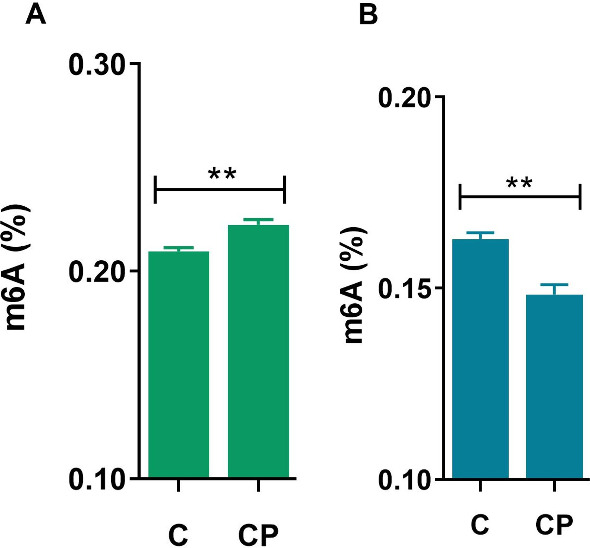
Global m^6^A methylation in HCC1937 and MDA-MB-231 cells exposed to carboplatin. Significantly global m^6^A methylation changes were observed in both cell lines with colorimetric measurement. (A) HCC1937 cells, (B) MDA-MB-231 cells. Statistical significance was determined using a *t* test, with results indicated by asterisks (** *p* < 0.01). C: control; CP: carboplatin.

### Survival Analysis across All Methylosome Genes

We analyzed the prognostic significance of METTL3, METTL14, WTAP, FTO, and YTHDF2 in 424 patients using the selected parameters. According to the Kaplan–Meier application, METTL3 (*p* = 0.00023) and FTO (*p* = 0.019) showed potential prognostic significance in TNBC cells. High expression of METTL3 appears to be associated with better prognosis, while low expression of FTO is associated with better prognosis (HR). These analyses are exploratory, but useful regarding the clinical relevance of m^6^A regulators in TNBC patients treated (Figure 6).

**Figure 6:**
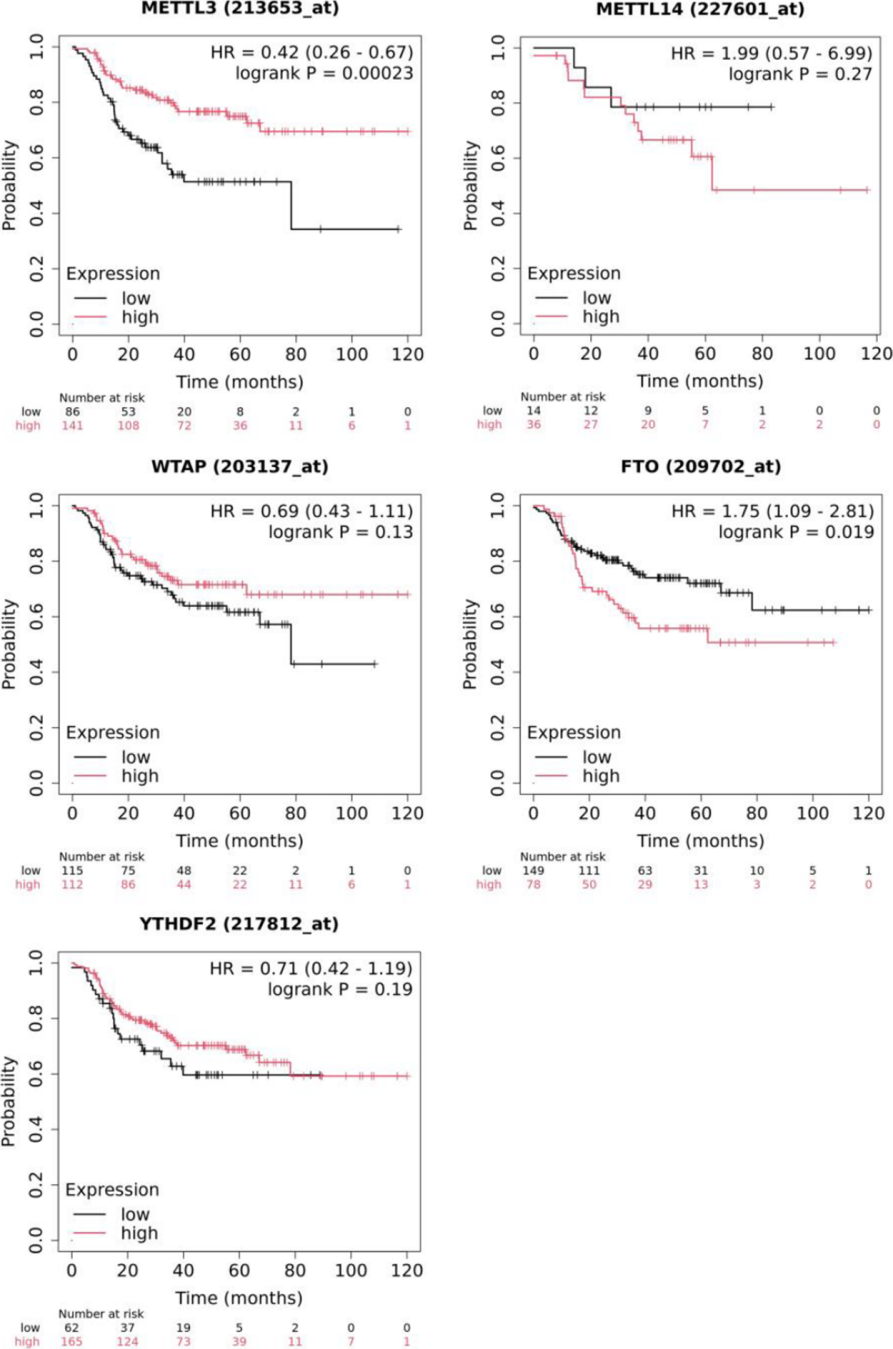
Survival analysis with negative receptors and chemotherapy and methylosome gene expression. RFS of breast cancer patients with negative receptor status and chemotherapeutic treatment correlated with methylosome gene expression based on the Kaplan–Meier plotter database. HR, hazard ratio.

### Molecular Docking Studies of CP in Methylosome Proteins

Molecular docking studies with the methylosome-associated proteins indicated that FTO and METTL3 exhibited the strongest predicted binding affinities for CP, with docking scores significantly surpassing those of their respective reference inhibitors. METTL3 had a docking score of −8,268, higher than that of adenosine (DS −7,553), while FTO’s score was −9,617, exceeding that of 6MK (DS −8,259). This enhanced binding may be attributed to the organic region of CP anchoring at the same site as the inhibitors, with the platinum moiety contributing additional stability, as illustrated in Figure 7. For METTL14, the docking score for CP (DS −5,841) was comparable to that of the reference inhibitor metformin (DS −5,917) and lower than eltrombopag (DS −7,852). For WTAP (DS −4,871) and YTHDF2 (−4,167), CP did not significantly outperform the inhibitors PG490 and phenylpyrazol CK-75, which had docking scores of −7,982 and −9,735, respectively, suggesting limited inhibitory potential of CP for these proteins.

**Figure 7:**
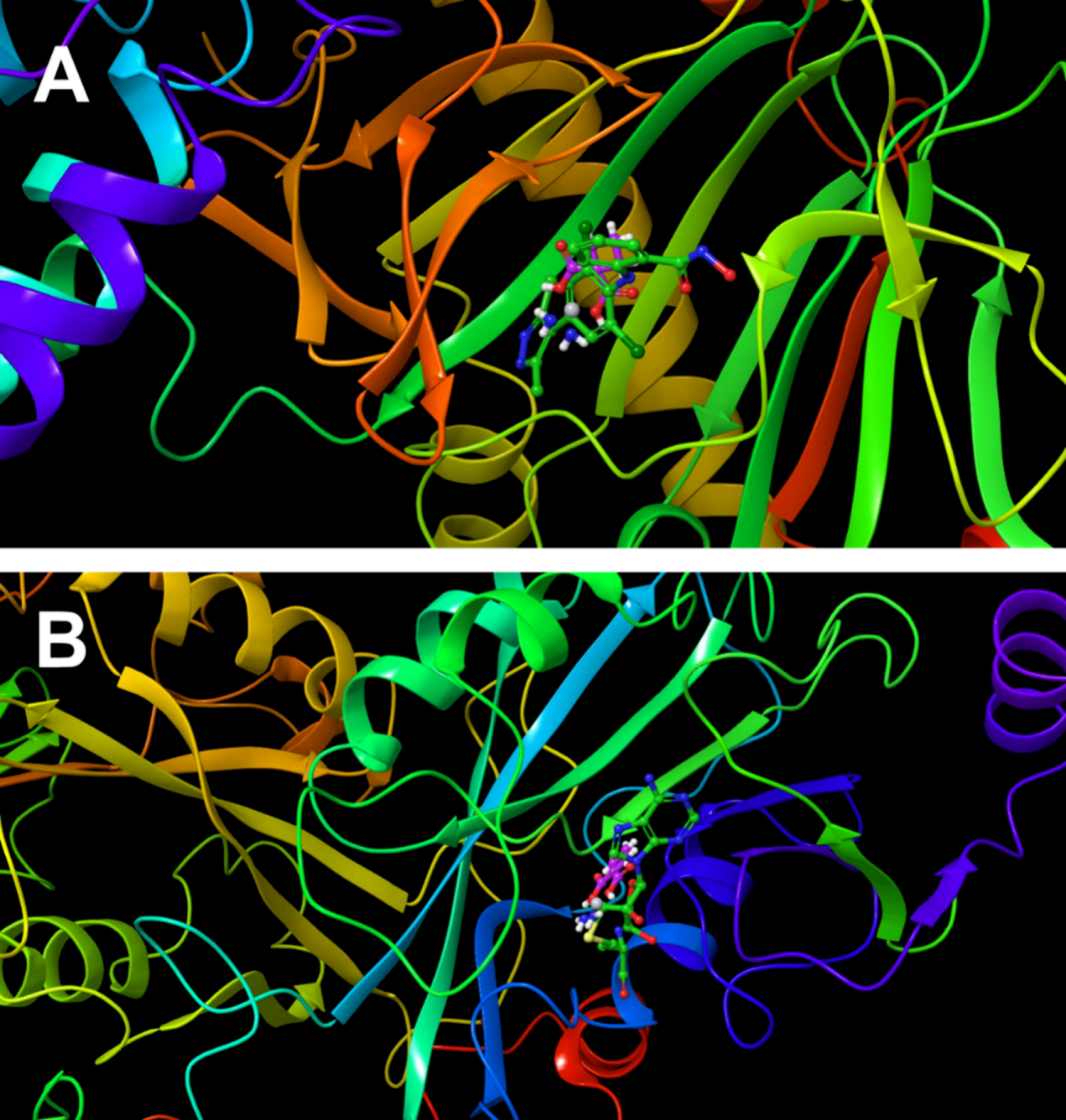
Predicted molecular docking of CP with FTO and METTL3. CP binding coupling with methylosome proteins, overlapping at the catalytic site with respect to the reference inhibitors, respectively: (A) FTO, (B) METTL3. in Schrödinger software obtained by Glide Extra Precision.

To determine the stability of the complex between the CP and the proteins of greatest interest, a molecular dynamics calculation was performed. In Figure 8, we observe that this complex is more stable with FTO, whereas with METTL3 it is slightly less stable, with a higher RMSD at later times. This is due to the large number of loops in the chain, characterized by residues between 100 and 150, as observed in the RMSF up to 6 Å. The gyration radius values remain constant.

**Figure 8:**
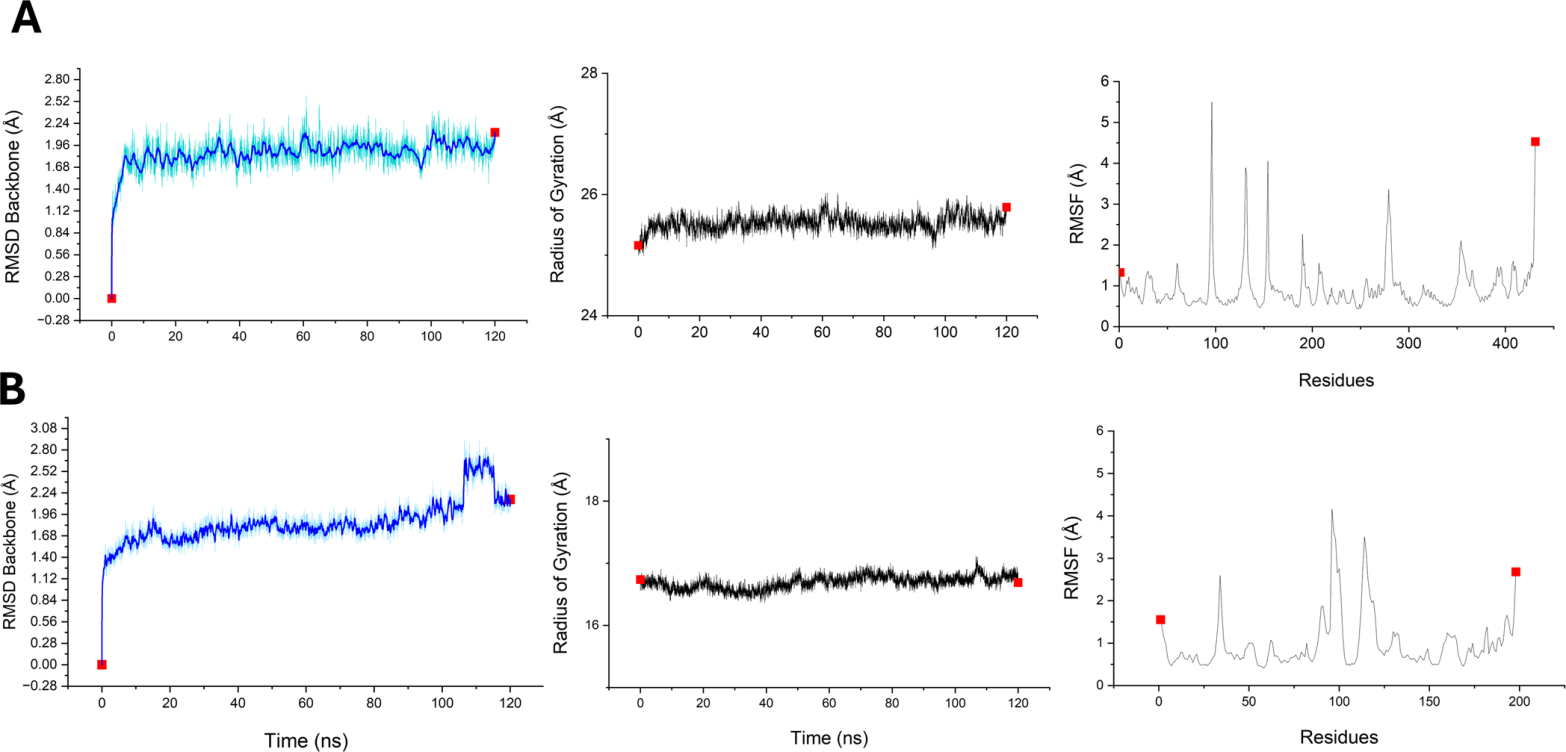
Molecular dynamics simulation parameters for the CP-FTO and CP-METTL3 complex. (A) FTO; (B) METTL3. MD simulation analyses showing root-mean-square deviation (RMSD), radius of gyration, and residue fluctuation (RMSF) of CP with methylosome proteins at 120 ns.

## Discussion

The median survival of patients with TNBC, which is an aggressive cancer, is 8 to 13 months. Survival is affected by several factors, such as older age, comorbidities, number of metastatic sites, histology, and treatment type.^18^ Unfortunately, despite the implementation of new therapeutic approaches, such poly­(ADP-ribose) polymerase/programmed death (PARP/PD-(L)­1/PD-1) inhibitors, the median 5-year survival rate has improved only slightly.^19,26,27^

Gene expression is frequently altered in different cancer types, a process that leads to progression and finally metastasis of tumor cells. RNA modifications, known as epitranscriptomic modifications, have recently been considered mechanisms by which tumor cells develop resistance to treatments. m^6^A regulates processes that include translation, splicing, export, stability, translation, and degradation of RNAs.^28^ As Hodara et al. pointed out, m^6^A is now recognized as a driver of therapeutic resistance. They found that enzalutamide-resistant prostate cancer cells have a different m^6^A profile than enzalutamide-sensitive prostate cancer cells.^29^

It is known that sensitivity and resistance are cell-type-dependent, so in this study, we evaluated the response to CP. The IC_50_ of CP was lower in HCC1937 cells (59 μg/mL) compared to MDA-MB-231 cells (125 μg/mL). According to Tassone et al., this difference may be due to the homozygous mutation in BRCA1 found in HCC1937 that affects the sensitivity and reduces the cells’ capacity to repair DNA.^30^

Dysregulation of RNA modifiers is common in cancer, so we further evaluated the post-treatment effects of CP on transcript and protein levels. METTL3 was up-regulated in HCC1937 cells (1.38) and down-regulated in MDA-MB-231 cells (0.62); these results agreed with the observed changes in global m^6^A levels. Some studies suggest that METTL3-mediated RNA m^6^A modification is involved in chemoresistance and leads to poor prognosis in cancers, such as bladder, ovarian, intrahepatic cholangiocarcinoma, or endometrial carcinoma. In these cancer types, it has been found that m^6^A addition by METTL3 allows reader proteins to recognize and prevent degradation of genes, such as TNFAIP3, TRIM47, NRF2, and FGD5-AS1. This process results in up-regulation of key genes, such as TGF-β, NF-κB, or PD-1/PD-L1, which leads to drug insensitivity or immune evasion.^31–34^ Down-regulation of METTL3 might restore chemosensitivity, but METTL3 activity is regulated by another m^6^A methyltransferase, METTL14, whose activity is required to maintain m^6^A homeostasis.^35^ Diverse evidence has shown that METTL14 can induce resistance to carboplatin, oxaliplatin, and cisplatin by affecting the processing of miRNA maturation or transcription factors expression in nonsmall cell lung cancer, colorectal, breast cancer T47D, and gastric cancer cell lines.^36–39^ In this work, CP was associated with an increase in METTL14 transcripts in MDA-MB-231 (1.9) but not in HCC1937 cells, in which the transcripts decreased (0.81). Unlike METTL3, this change did not show any relationship with m^6^A levels. Recently, Zhang et al. found that the overexpression of METTL14 in chronic myeloid leukemia facilitates cancer progression by altering sensitivity to imatinib.^40^

WTAP promotes the formation of the WTAP/METTL3/METTL14 methyltransferase complex and leads to an increase in mRNA stability. Tan et al. found that oxaliplatin treatment in colorectal cancer cells causes an increase in the m^6^A mark and WTAP protein expression and that WTAP depletion produces an increase in sensitivity to oxaliplatin in an m^6^A -dependent PANoptosis manner through NRF2.^41^ WTAP overexpression promotes cisplatin resistance, colony formation, and apoptosis decrease in ovarian cancer cells and knockdown inhibited all those conditions, according to the results by Hong et al.^42^ Wang et al., analyzing the high-grade serous cancer subtype of ovarian cancer, found that down-regulation of WTAP provides an appropriate metabolic condition for the repair of tumor cells damaged by platinum treatment and down-regulation of m^6^A.^43^ We found the opposite of WTAP expression in the selected breast cancer cell lines; in HCC1937 cells, it was down-regulated (0.48), but up-regulated in MDA-MB-231 cells (1.35), so we could determine the variability between different breast cancer cell lines. According to several studies, changes in WTAP expression can lead to reprogramming of the stability of various genes’ messengers, including transcription factors, by modifying the m^6^A levels. As Ping et al. suggest, this modification may occur due to the absence of WTAP, which causes the RNA-binding capability of METTL3/METTL14 to significantly decrease.^44^

Two methylosome components, YTHDF2 and FTO, that were analyzed in this study had the same behavior between lines. YTHDF2 was up-regulated, and FTO was down-regulated. YTHDF2, an m^6^A reader, selectively recognizes and shuttles m^6^A-modified RNAs for degradation by multiple mechanisms.^45^ Low YTHDF2 expression has been associated with a better prognosis in lung adenocarcinoma, which responds better to PD-1/PD-L1 inhibitor treatment.^46^ In intrahepatic cholangiocarcinoma, YTHDF2 is up-regulated in cisplatin chemoresistant tissues, which leads to DNA damage inhibition via downregulation of CDKN1B, a cyclin-dependent kinase inhibitor.^47^ FTO overexpression has been associated with chemoresistance in colorectal cancer based on tolerance to 5-FU treatment, and subexpression of FTO by Rhein inhibitor was found to restore sensitivity.^48^ In our study, FTO was down-regulated after CP treatment in a concentration-dependent manner in both TNBC cell lines, similar to some other methylosome components. We found that transcripts and protein levels were similar in HCC1937 cells, thus further confirming protein expression; however, in MDA-MB-231 cells, we observed a decrease, which could have been because of CP on post-transcriptional processing in those cells. Pietras et al. reported that platinum drugs affect protein translation.^49^ Our study also found that the m^6^A methylation mark presented a different behavior between cell lines. HCC1937 showed an increase in this mark after CP treatment, a finding that is supported by METTL3 up-regulation and FTO down-regulation. In MDA-MB-231 cells, the m^6^A marks were reduced after CP treatment as a result of a decrease in METTL3. As observed, the interaction between the methylosome components is complex, and the m^6^A methylation mark increases or decreases depending on this interaction. METTL3 expression was decisive for the levels of m^6^A, regardless of the behavior of the other components and appears to be a determinant for survival. It is known that more m^6^A marks on mRNAs will determine YTHDF2 promotion of greater messenger degradation, a process that has an impact on resistance to chemotherapy drugs.

When we performed the bioinformatic analysis relating the levels of methylosome component expression to the survival of patients who had undergone treatment, we found that survival was linked to an increase in METTL3 levels (*p* = 0.00023) and a decrease in FTO expression (*p* = 0.019), while the levels of METTL14, WTAP, and YTHDF2 were not significant. However, it was not possible to obtain the patients’ chemotherapy regimens; thus, it remained unclear whether the CP treatment observed in vitro would be the same as in patients treated with CP. Our docking analysis predicted that CP has the capability to modulate FTO by showing the most stable protein–CP interaction, so this effect is directly derived intuitively. The presence of this FTO modulation, together with changes in METTL3, suggests possible regulation of mRNA and protein after the treatment, as observed in the experiments. Our in silico findings provide a robust structural rationale for the experimental biological effects observed after CP treatment. The initial docking analysis predicted favorable binding affinities for CP toward both FTO and METTL3, suggesting a potential dual-regulatory mechanism on the m^6^A methylation machinery that aligns with the observed alterations in downstream mRNA and protein expressions. By extending these static models into atomistic MD simulations, we confirmed that both complexes maintain structural integrity over time. While the FTO–CP complex exhibited remarkable stability with RMSD values consistently under 2.2 Å, the late-trajectory fluctuation observed in METTL3 (shifting from 1.96 Å to 2.5 Å after 100 ns) was mapped to the intrinsic flexibility of its loop domains (residues 100–200) rather than a destabilization of the binding pocket. These dynamic and structural insights reinforce the hypothesis that CP can effectively target and modulate key epitranscriptomic regulators.

Our study has several limitations. Additional biochemical assays (such as enzymatic activity assays or isothermal titration calorimetry) and metallodrug-specific modeling are needed to confirm the direct binding and inhibitory kinetics of CP on methylosome components. We acknowledge that bioinformatic analysis is exploratory and should be interpreted with caution.

## Conclusions

Factors such as platinum-based drugs, drug concentration, exposure time, and cancer cell type may affect the expression of genes involved in the m^6^A regulation. Based on our findings, we hypothesize that CP mainly affects the expression of FTO and METTL3, which may be associated with patient survival. However, due to the complex interactions between methylosome components, these changes affect cell lines and m^6^A levels in different ways, closely involving the reader function of YTHDF2. This process may be associated with the dysregulation of genes involved in the tumor phenotype and drug resistance.

## Supplementary Material



## Data Availability

Data sharing does not apply to this article as no data sets were generated or analyzed during the current study.

## Notes

Inhibitors for methylosome complex components (PDF)

## Abbreviations

CP: carboplatin
TNBC: triple-negative breast cancer
m^6^A: N6-methyladenosine
METTL3: methyltransferase-like 3
METTL14: methyltransferase-like 14
WTAP: Wilms tumor 1-associating tumor
FTO: fat mass and obesity-associated protein
YTHDF1,2: RNA-binding proteins
