# Supplementary material for: Carboplatin Is Associated with Changes in Components of the m6A Machinery in Triple-Negative Breast Cancer Cells
Source: ACS Omega. 2026 Jul 7;11(28):41747–57. doi: 10.1021/acsomega.6c01546 (PMC13393194; doi:10.1021/acsomega.6c01546)
Supplement: Supplementary file 1 [file ao6c01546_si_001.pdf]

Carboplatin is associated with changes in components of the m^6A machinery in triple-negative breast cancer cells.

Ricardo Villalobos-Valencia [1]‡, Carlos R. Alvizo-Rodríguez [2]‡, Alan Carrasco-Carballo [3], José A. Sierra-Ramírez [1], Uriel López-Vázquez [1,2], Emmanuel Seseña-Méndez [4], and Marta E. Hernández-Caballero [2].*

1 Sección de Estudios de Posgrado e Investigación, Escuela Superior de Medicina, Instituto Politécnico Nacional, Ciudad de México, México

2 Laboratorio de Biología del Cáncer, Facultad de Medicina, Biomedicina, Benemérita Universidad Autónoma de Puebla, Puebla, México.

3 Secretaría de Ciencia, Humanidades, Tecnología e Innovación, Laboratorio de Elucidación y Síntesis en Química Orgánica, Herbario y Jardín botánico, Vicerrectoría de Investigación y Estudios de Posgrado, BUAP, Puebla, México.

4 Laboratorio de Neuroinmunologia, Facultad de Medicina, Biomedicina, Benemérita Universidad Autónoma de Puebla, Puebla, México.

‡ Equal contribution

** Correspondence: elena.hernandezcab@correo.buap.mx*

Table S1. Inhibitors for methylosome complex components

Gene	Inhibitor	Reference
METTL3/METTL14	metformin, eltrombopag	https://doi.org/10.1016/j.ejmech.2025.117560
WTAP	PG490	https://www.jci.org/articles/view/177932
YTHDF2	phenylpyrazol CK-75	https://doi.org/10.1021/jacsau.4c00754
FTO	meclofenamic acid (MA)	https://doi.org/10.1016/j.neuropharm.2025.110462
